# Mandibular overdenture with a single implant in the canine region (c-SIMO): a feasibility study

**DOI:** 10.1007/s00784-024-05723-1

**Published:** 2024-05-21

**Authors:** Sabrina Maniewicz, Thalita Fernandes Fleury Curado, Murali Srinivasan, Cláudio Rodrigues Leles, Frauke Müller

**Affiliations:** 1 Division of Gerodontology and Removable Prosthodontics, University Clinics of Dental Medicine, University of Geneva, Geneva, Switzerland; 2 Department of Oral Rehabilitation, School of Dentistry, Federal University of Goias, Goiania, Brazil; 3 Clinic of General-, Special Care and Geriatric Dentistry, Center for Dental Medicine, University of Zurich, Zurich, Switzerland; 4 Department of Reconstructive Dentistry and Gerodontology, School of Dental Medicine, University of Bern, Bern, Switzerland; 5 Division of Geriatrics, Department of Rehabilitation and Geriatrics, University Hospitals of Geneva, Thônex, Switzerland

**Keywords:** Implant overdenture, Single-implant overdenture, Dental prosthesis, Pilot study

## Abstract

**Objectives:**

The aim of this multi-center pilot study was to assess the viability and feasibility of a novel treatment concept – the canine-positioned single implant mandibular overdenture (c-SIMO), with the single implant placed on the patient's preferred chewing side instead of the midline.

**Materials and methods:**

Participants received a single implant in the canine region of their preferred chewing side, based on an Asymmetry Index observed during mastication. The pre-existing mandibular denture was transformed into a c-SIMO on a spherical attachment. The primary outcome was oral health-related quality of life (OHRQoL), measured with GOHAI and OHIP-EDENT. Secondary outcomes included denture satisfaction index (DSI), chewing efficiency (CE), maximum bite force (MBF), implant survival and success, and prosthetic maintenance. Data analysis included descriptive statistics and bivariate comparison tests.

**Results:**

Fifteen participants received the c-SIMO treatment (mean age: 69.9 ± 7.0). Implant success and survival rates were 100% at 1 year. Patient-reported outcome measures improved significantly compared to pre-treatment values (OHIP-EDENT: *p* = 0.001; DSI: *p* = 0.001; GOHAI: *p* = 0.002). Masticatory outcomes also improved significantly (CE: *p* = 0.001; overall MBF: *p* = 0.005). Post-implant, MBF was significantly higher in the ipsilateral side compared to the contralateral side at 2 weeks (*p* = 0.019) and 3 months (*p* = 0.015), but no longer at T3 (*p* = 0.730). Common prosthodontic events included denture base adjustments (*n* = 17) and matrix activation (*n* = 9).

**Conclusions:**

This pilot study concludes that c-SIMO is a promising treatment option, and a potential alternative to the single midline implant overdenture.

**Clinical relevance:**

The novel treatment concept of a canine-positioned single implant mandibular overdenture could be a viable treatment alternative to the midline positioning.

## Introduction

Although it is widely accepted by the scientific community that using two implants in the mandible is the preferred method for ensuring the stability and retention of a mandibular complete denture, other treatment alternatives may be considered [30, 67]. The choice of different treatment strategies may vary not only internationally and regionally, but will also vary individually, due to a multitude of both operator- and patient-related reasons [31]. Many individuals who have lost all their teeth are still rehabilitated with a conventional complete denture. Low economic resources, the invasiveness of the intervention as well as general health issues are frequent barriers for elderly and fragile patients concerning dental implants or implant-related treatment options [45, 51, 72]. If economic barriers restrict treatment options, then it is imperative to consider affordable treatment alternatives. Furthermore, minimally invasive treatment approaches are one of the cardinal objectives of optimal surgical treatment today, especially in frail and geriatric patients. Minimizing the number of implants required for dental treatment remain within the spectrum of minimally-invasive treatment concepts and will help invariably reduce surgical complications and morbidity, treatment burden, patient and operator stress, postoperative complications and/or morbidity, and finally, cost [61].

A single implant placed in the midline of the mandible to retain a mandibular denture (single implant mandibular overdenture; SIMO) is a proven treatment concept for edentulous patients [16, 39, 54, 59, 62, 66, 71]. The benefits of this treatment protocol are multiple [2, 5–8, 16, 17, 20, 22, 24, 25, 39, 41, 52, 54, 57, 59, 62, 66, 71]. Observational studies in the literature have evaluated the performance and success of the SIMO concept with regard to the biologic success of the implant supporting the reconstruction, the rate of prosthodontic complications and the need for maintenance. Most studies comparing SIMOs directly to 2-IODs have demonstrated no differences in implant survival rates between both modalities [2, 5, 6, 16, 59, 66, 71]. Studies have reported that the frequent prosthodontic complications and maintenance events encountered with SIMOs included denture fracture around the midline implant and matrix activation or replacement, respectively [5, 16, 20, 22, 24, 25, 41, 52, 59, 71]. Furthermore, SIMOs have been evidenced with increased patient satisfaction and improved Oral Health-Related Quality of Life (OHRQoL) [5, 7, 8, 16, 17, 20, 52, 57, 71]. Although only a limited number of studies have objectively examined and quantified masticatory function, reports show that both masticatory efficiency and bite force increase along with a positive influence on the muscular activity and chewing patterns in patients rehabilitated with SIMOs [7, 8, 18, 37, 43, 56].

An important aspect to consider is that the choice of the implant positioning in the mandibular midline is not based on evidence demonstrating its superiority compared to an alternative position, which could present similar or improved results. Chewing support, whether provided by teeth or by implants, is most effective when the support is closest to the chewing center, located in the second premolar – first molar region [35]. The closer the support to this area, the more effective the support from the abutment. Hence, positioning the implant closer to the chewing center might significantly increase the chewing efficiency (CE) as well as the maximum voluntary bite force (MBF). Therefore, a valid hypothesis would be that if the single implant was placed in a more lateralized position in the mandible on the patients’ preferred chewing side rather than the midline, then it could potentially improve the masticatory outcomes even further with a SIMO.

The use of a single implant positioned in the canine region (c-SIMO) instead of in the midline could further enhance the performance of SIMOs, particularly concerning prosthodontic drawbacks. The relatively high denture fracture rates reported in previous studies may be attributed to a fulcrum effect that the midline implant might have when posterior occlusal forces act on the overdenture. Furthermore, the available prosthetic volume for incorporating the attachment housing into the denture is limited, and therefore at higher risk of developing cracks and fractures during posterior functional loading. By placing the implant in a more lateralized position, firstly the fulcrum effect is eliminated thus potentially reducing the fracture risk of the denture base, and secondly there is more prosthetic volume in the denture to accommodate the retentive element and the corresponding housing which further minimizes risk for fracture. Another important benefit in the canine positioning of the single mandibular implant is the future possibility of adding another implant in the contra-lateral canine region, to convert the existing c-SIMO to the gold-standard two-IOD, if requested.

The aim of this pilot study was therefore to determine whether the novel treatment concept of stabilizing a mandibular complete denture with a single implant placed in the canine region (c-SIMO) of the patient’s preferred chewing side is a viable and feasible treatment option, by assessing implant and prosthetic survival/success, masticatory function (CE), OHRQoL and patient satisfaction.

## Materials and methods

This multi-center, single-arm pilot study is reported with adherence to the CONSORT extension to pilot and feasibility trials and the STROBE statement, as far as applicable, and according to the available guidelines for non-randomized studies [28, 44, 70]. The trial was approved by the relevant ethical committees of each research centers (Swiss centers: 2020–01780; Brazilian center: CAAE 39165920.1.0000.5083). Written and informed consent was obtained from all the included participants.

### Participants

Participants were recruited from the patient pools of the University Clinics of Dental Medicine of the University of Geneva, Switzerland, the Center for Dental Medicine of the University of Zurich, Switzerland, and the School of Dentistry of the Federal University of Goias, Brazil. Participants were screened regarding the inclusion and exclusion criteria listed in Table 1, and the preferred chewing side was determined.

**Table 1:** Inclusion and exclusion criteria for participant recruitment

Inclusion criteria
• 55 years or older • Willing to participate and sign an informed consent • Completely edentulous participants who live independently and are not dependent for care • Participants rehabilitated with maxillary and mandibular conventional complete dentures considered sufficient or ones that can be rendered sufficient via reline and/or renewal of the prosthetic teeth • Healed edentulous mandible (minimum 6–8 weeks since last extraction in the anterior zone (4–4) and one year in the posterior zone (5–8)) • Physical status of ASA1 or ASA2*
Exclusion criteria
• Contraindications to the medical devices used, e.g. known hypersensitivity or allergy • Inability to perform adequate oral hygiene • Incapability to provide written informed consent and compliance to the protocol • History of repeated unjustifiably missed appointments • Surgical risk factors such as, but not limited to, uncontrolled diabetes, immunosuppression, radiation, chemotherapy, or antiresorptive medication (ex. bisphosphonates) • Heavy smoking habit of > 20 cigarettes per day • Moderate/severe dementia^†^ or depression^‡^ • Xerostomia with less than 0.7 ml/min of Stimulated Salivary Flow Rate (SSFR)^§^ • Reported severe bruxism or clenching habits, clinically present oro-facial pain • Incorporated metal framework in any of the complete dentures • Excessive occlusal wear of the denture teeth with loss of more than 1/3 of cuspid height or worn denture resin
Post-hoc exclusion criteria
• Ridge dimensions less than 6 mm (width) by 10 mm (height) in the canine area • Ridge defects requiring bone augmentation procedures

*ASA Physical Status Classification System [1]

^†^ Mini Mental State Examination [33]

^‡^ Geriatric Depression Scale [63]

^§^ Stimulated Salivary Flow Rate of whole saliva [27]

### Intervention and protocol

**Fig. 1:**
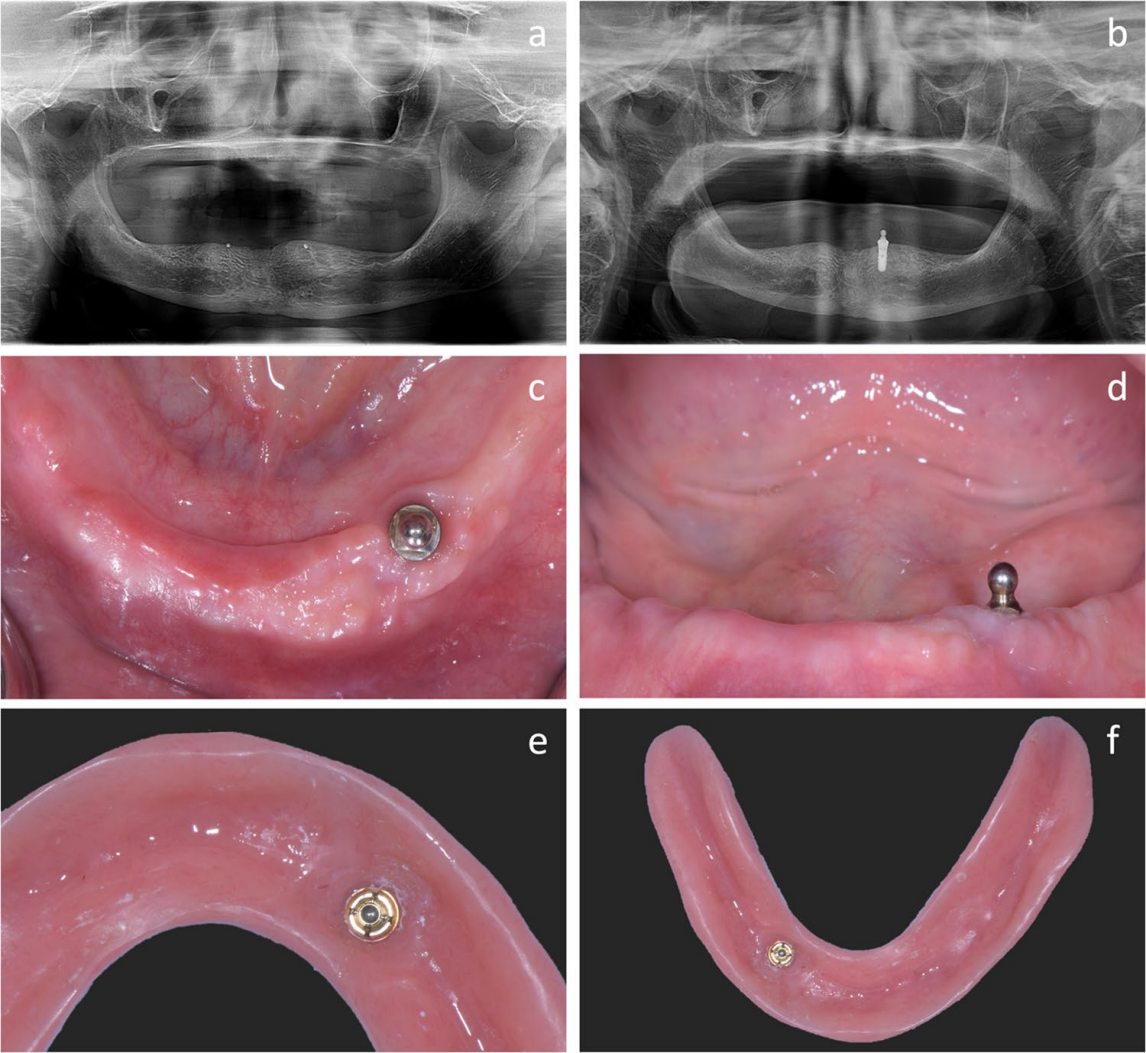
**a** Baseline OPT with gutta-percha markings for spatial referencing; **b** OPT after 1 year with normal peri-implant bone levels; **c** and **d** Occlusal and frontal view of spherical patrix on implant 33, **e** Close-up of elliptical matrix; **f** Denture base with incorporated matrix

Prosthetic and surgical diagnostics were completed in a preoperative visit with thorough examination of the patient’s medical and dental history, as well as a clinical examination including the determination of the preferred chewing side. A radiological assessment was performed with an Orthopantomogram (OPT). The existing mandibular denture was marked with gutta-percha dots in the canine regions prior to image-taking for the purpose of spatial reference. The dentures were stabilized during image-taking with cotton rolls placed in between the upper and lower posterior teeth, to preclude denture displacement during the biting on the anterior fork of the OPT machine (Fig. 1a).

Implant surgery was performed under local anesthesia, with a mid-crestal incision and elevation of a minimal mucoperiosteal flap. The incision was further extended crestally or with releasing incisions where ridge flattening or ridge defects or proximity of sensitive anatomical structures (e. g. the mental foramen) required a more extended overview. The existing denture was used as a surgical template and the implant surgery was performed according to the manufacturer’s instructions. Participants received one 4.1 × 8 mm Straumann Standard Plus Regular Neck implant (Institut Straumann AG, Basel, Switzerland). The implant was placed either in the tooth position of 33 or 43, depending on the participant’s preferred chewing side. Primary implant stability was measured immediately after insertion. The appropriate healing abutment was placed on the implant and the flap was sutured allowing for transmucosal healing whenever applicable. The existing denture was adapted for unloaded wound healing. The implant placement was verified with a post-operative OPT or intraoral radiograph. Post-surgical recall visits were scheduled at 7 to 15 days after implant placement for suture removal and denture adaptation or upon request.

Following an early loading protocol, participants were invited to return 6 to 8 weeks after implant placement, at which timepoint the implants were loaded by placing a spherical retentive anchor (Spherical retentive anchor; Institut Straumann AG, Basel, Switzerland) and a corresponding elliptical matrix with a rotational activation system (Swiss centers: Dalbo-PLUS, Cendres + Métaux SA, Biel, Switzerland; Brazilian center: Elliptical matrix; Institut Straumann AG, Basel, Switzerland) [34] (Fig. 1b-d). Depending on the clinical situation, the attachment housings were either directly incorporated using a pick-up technique with self-curing acrylic resin or indirectly processed in the pre-existing prosthesis following a reline wash-impression with polyether impression material (Impregum® Garant® L Duosoft®; 3 M Company, Saint Paul, MN, USA); the transformed prosthesis was delivered on the same day (Fig. 1e-f). Matrices were activated until adequate retention was obtained. Recall visits were scheduled as stipulated by the trial protocol. Additional adjustments and repairs were performed as required.

### Outcome measures

The outcome measures were recorded at the following pre-determined timepoints: baseline (BL) before intervention, T_1_ (2 weeks after IOD insertion), T_2_ (3 months after IOD insertion) and T_3_ (1 year after IOD insertion).

#### Preferred chewing side

The preferred chewing side (PCS) was determined by using the method described by Mizumori and co-workers [48]. This method involves a calculation of the Asymmetry Index (AI), using the formula:$\mathrm{AI}=\frac{number\;of\;right\;chewing\;strokes-number\;of\;left\;chewing\;strokes}{number\;of\;right\;chewing\;strokes+number\;of\;left\;chewing\;strokes}\times100\%$ . AI was recorded via a video camera during mastication of a chewing-gum during 20 chewing cycles. In cases with a low masticatory laterality (AI < 30%), the preferred chewing side was determined according to the participants’ stated PCS using a Visual Analog Scale [32]. PCS was assessed at all time points.

#### Implant survival and success: clinical and radiographical outcomes

Implant success, survival and failure was determined using the Health Scale for Dental Implants [47], a scale which takes into account indices comprising pain, mobility, radiographic crestal bone loss, probing depths and peri-implant exudate. Peri-implant conditions were classified using the modified Plaque Index (modPI) and the modified Bleeding Index (modBI) [49]. The pocket probing depth (PPD) was measured in mm from the peri-implant mucosal margin to the bottom of the sulcus or pocket, while the width of the keratinized tissue, also measured in mm, was measured at the buccal and lingual side. Peri-implant clinical parameters were assessed at T_1_, T_2_ and T_3_.

Radiographical crestal bone levels were assessed on the digital OPTs [13, 15]. Reference markings in the form of parallel lines were made on all radiographs using reproducible reference points on the implants (implant shoulder and apex), as well as markings at the bone level on the mesial and distal side of the implant (Adobe Photoshop Elements 2.0; Adobe Systems Inc, San Jose, CA, USA). The distances between the reference markings and the crestal bone levels were calculated using an image analysis freeware to account for any distortion present (ImageJ, V1.54, National Institutes of Health). A single investigator (SM) performed these measurements, which were carried out at T_3._

#### Chewing Efficiency (CE)

CE was evaluated with a two-color mixing ability test [60]. Participants were given a validated two-colored chewing gum and were requested to chew the specimen for 20 chewing cycles (Goiania and Zurich centers: Hue-Check Gum, Orophys GmbH, Muri b. Bern, Switzerland; Geneva center: Gum for 8020 Promotion Foundation, Lotte, Tokyo, Japan). The resulting bolus was evaluated visually (subjective assessment). The gum was then flattened to a wafer thickness of 1 mm, digitized, and opto-electronically analyzed [Variance of Hue (VOH)] using a purpose-built software (ViewGum, dHAL Software, Kifissia, Greece) [38]. Chewing efficiency was assessed at all time points.

#### Maximum Bite Force (MBF)

MBF was measured in Newtons using a digital force gauge (Swiss centers: Occlusal Force-Meter GM 10®, Nagano Keiki Co. Ltd., Tokyo, Japan; Brazilian center: DMD® Kratos, Kratos Equipamentos Industriais Ltda, Cotia, Brazil) placed in the first molar area with a stabilizing block of the same thickness (8.7 mm and 14.6 mm for the Occlusal Force-Meter GM 10® and the DMD®, respectively) on the contralateral side to avoid dislodgement of the prostheses. The participants were encouraged to use their maximum strength to bite on the instrument until a measurement was effectively recorded. Three recordings were carried out per side, and the mean of the highest value registered for each side was used for analysis. MBF was measured at each time point.

#### Denture Satisfaction Index (DSI)

Denture satisfaction was evaluated using a 100 mm visual analog scale (VAS)-based questionnaire [11]. It is used to evaluate “comfort, ability to chew, stability, esthetics, ability to speak and ease of cleaning” [11]. Participants were trained in filling out this type of VAS beforehand. Denture satisfaction was evaluated at all time points.

#### Oral Health-Related Quality of Life (OHRQoL)

The General Oral Health Assessment Index (GOHAI) was used to assess the impact of oral disorders on OHRQoL via a 12-statement Likert-format questionnaire [10, 26, 40, 68]. A shorter version of the 49-item Oral Health Impact Profile (OHIP) questionnaire that was specifically developed for edentulous persons, was also used to assess the OHRQoL [3, 4, 64, 65]. The OHRQoL is considered good when the GOHAI score is high, or when the OHIP-EDENT score is low. These instruments were filled independently by the participants at all time points.

#### Prosthetic survival and success: maintenance and complications

The prostheses were examined for any complications according to the criteria proposed by Brägger in the ITI Treatment Guide Volume 8 [14]. This includes complications concerning the attachment components, the overdenture itself, and the denture teeth. This assessment was carried out at all timepoints, as well as additional unscheduled timepoints when necessary.

### Statistical analysis

A target sample of fifteen participants completing the study was considered adequate for obtaining sufficient preliminary data. Data analysis included descriptive statistics and bivariate comparison tests. Normal distribution of data was tested using the Shapiro-Wilks test (*p* < 0.05). The Wilcoxon signed-rank test was used for pairwise comparison of outcome measures between baseline (before intervention) and the follow-up assessments (2 weeks, 3 months, and 1 year) with the significance set to *p* < 0.05. The 1-year survival and success rates were recorded for the implants and the prostheses. The incidence rates of prosthodontic events were registered throughout the complete follow-up period. All statistical analyses were performed using Microsoft Excel and IBM-SPSS 24.0 softwares.

## Results

Between May 2021 and July 2022, sixteen participants were recruited for this pilot study. One participant had an early implant failure before loading and was subsequently excluded from the study. Therefore, a total of 15 participants received the overdenture treatment and were analyzed in the study. The characteristics of the participant pool are detailed in Table 2, including the preferred chewing side and the edentulous ridge classification with the Prosthodontic Diagnostic Index [46]. Nine participants were treated at the University of Goias (Brazil), five at the University of Geneva (Switzerland) and one at the University of Zurich (Switzerland). Nine implants (60%) were inserted on the right side and 6 (40%) on the left, according to the predetermined preferred chewing side (PCS). All participants completed the 1-year follow-up, and none requested the placement of an additional implant during the observation period.

**Table 2:** Participant demographics and characteristics

Mean age ± SD (years)	69.9 ± 7.0
Sex *N* (%)	-
Total	15 (100)
Women	11 (73.3)
Men	4 (26.7)
Preferred chewing side *N* (%)	-
Right	9 (60)
Left	6 (40)
None	0 (0)
Prosthodontic diagnostic index *N* (%)	-
Maxilla	-
Type A	11 (73.3)
Type B	4 (26.7)
Type C	0 (0)
Type D	0 (0)
Mandible	-
Type A	6 (40.0)
Type B	2 (13.3)
Type C	3 (20.0)
Type D	4 (26.7)
Type E	0 (0)

*N* Number; *SD* Standard deviation

Implant success and survival rates were 100% at T3. Table 3 summarizes the peri-implant findings, measured 2 weeks, 3 months, and 1 year after loading. Two weeks after loading, plaque accumulation was low (median = 0.84) and bleeding on probing was rarely observed (median = 0) and did not change significantly in all the subsequent follow-up periods. Similarly, probing depth and the width of keratinized mucosa remained unaltered throughout the study. Based on radiographic measurements of peri-implant bone loss, no or negligible changes were observed at T3 and, therefore, no data analysis were performed.

**Table 3:** Measurements of the peri-implant outcome variables at the follow-up visits (*n* = 15)

	Plaque	Bleeding	Probing depth	KMW
2 weeks (T_1_)	0.88 (1.6)	0.0 (0.56)	2.60 (0.81)	1.69 (2.50)
3 months (T_2_)	0.50 (1.5)^ns^	0.0 (0.75)^ns^	2.25 (0.50)^ns^	1.13 (2.25)^ns^
1 year (T_3_)	0.00 (2.0)^ns^	0.0 (0.50)^ns^	2.50 (0.75)^ns^	1.00 (2.25)^ns^

Results are presented as median (and interquartile range)

^ns^ Difference not significant – Wilcoxon signed-rank test (all tests compared to T1)

*KMW* Keratinized mucosa width [mm]

**Fig. 2:**
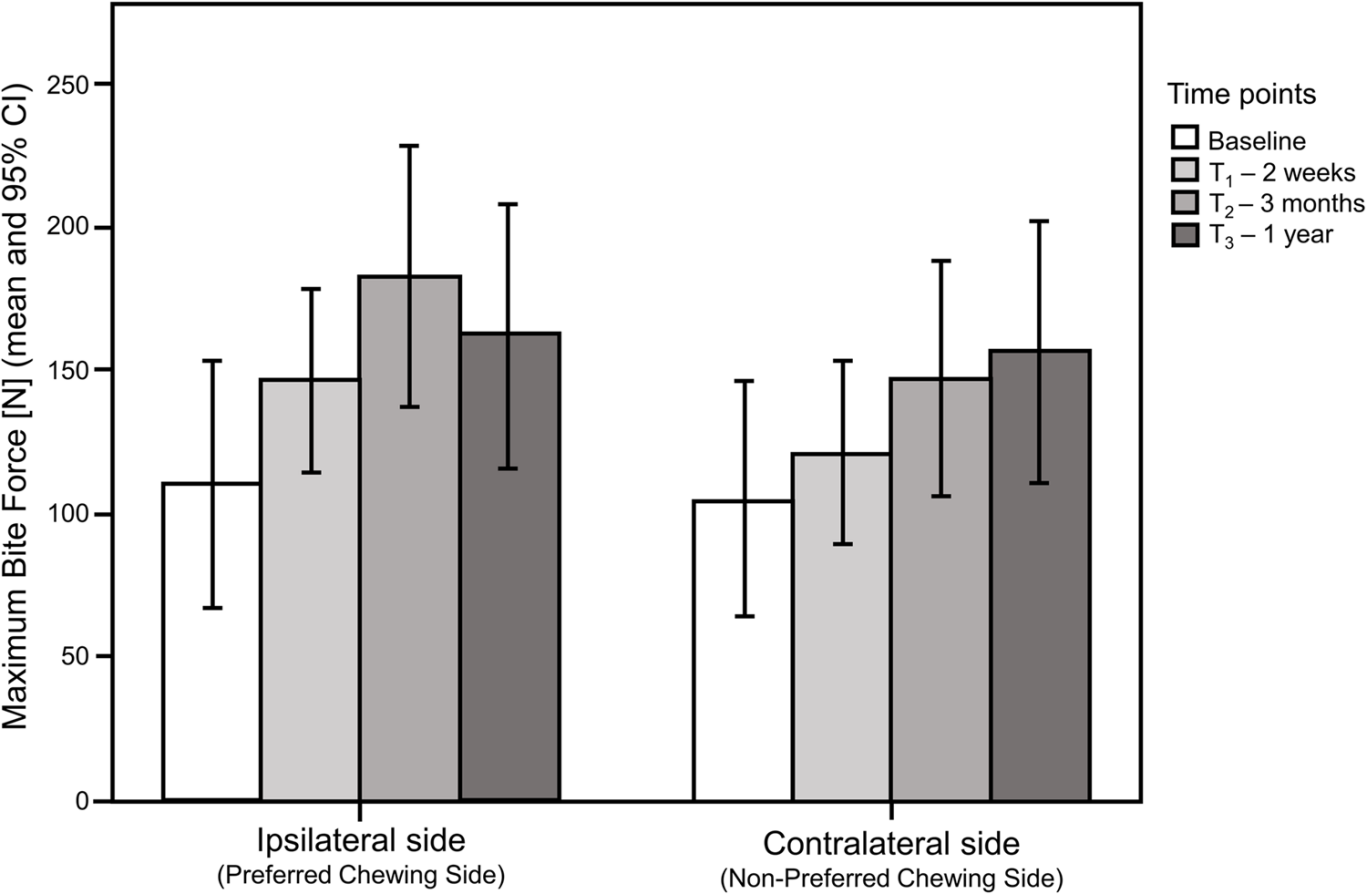
Maximum Bite Force for each chewing side at baseline and follow-up visits. Results are presented as mean (and 95% Confidence Interval)

The 1-year results concerning patient-reported and functional masticatory outcomes are detailed in Table 4. There was a significant improvement in all patient-reported outcome measures after one year compared to pre-treatment values, including OHIP-EDENT scores (*p* = 0.001), Denture Satisfaction Index (*p* = 0.001), and GOHAI scores (*p* = 0.002). The improvements in outcomes were significant at the first post-insertion visit after implant loading (2 weeks) and persisted until the 1-year follow-up. There was also a significant improvement in masticatory outcomes assessed at one-year follow-up compared to baseline values (chewing efficiency: *p* = 0.001; overall maximum bite force: *p* = 0.005) with the improvements being significant from 3 months of use onwards. The magnitude of changes at 1-year were considered large (effect size ≥ 0.50) for all patient-reported outcome measures (PROMs). Overall, the average pooled MBF from both sides changed from 107.6N ± 71.9 to 151.2N ± 64.1 in the pre- and post-implant stages, respectively (*p* = 0.008). The overall mean MBF in the post-treatment period were 161.9N ± 67.5 and 140.6N ± 63.6 for the ipsilateral and the contralateral sides, respectively (*p* = 0.016). In addition, significant increase in the MBF after the implant placement was observed for both the ipsilateral (*p* = 0.013) and contralateral (*p* = 0.004) sides. MBF had been similar in the ipsi- and contralateral sides at baseline (*p* = 0.470). The measurements of the MBF in the ipsilateral and contralateral sides according to the study time points are shown in Fig. 2. In the post-implant stages, MBF was significantly higher in the ipsilateral side compared to the contralateral side at T1 (*p* = 0.019) and T2 (*p* = 0.015), but no longer at T3 (*p* = 0.730).

**Table 4:** Measurements of the patient-reported and functional outcome measures at baseline and follow-up visits (*n* = 15)

	Patient-reported outcomes			Functional outcomes	
	OHIP-EDENT	Satisfaction	GOHAI	VoH	Overall MBF [N]
Baseline (BL)	28.0 (27.0)	67.9 (25.7)	36.0 (15.0)	0.65 (0.19)	80.4 (109.6)
2 weeks (T_1_)	5.5 (8.5)**	94.7 (8.9)**	57.5 (7.0)**	0.49 (0.37)^ns^	125.0 (63.8)^ns^
3 months (T_2_)	3.0 (5.0)**	98.4 (7.5)**	59.0 (7.0)**	0.36 (0.29)**	158.8 (121.1)**
1 year (T_3_)	2.0 (6.0)**	98.0 (3.8)**	59.0 (5.0)**	0.35 (0.29)**	136.0 (96.4)**
Mean difference (SD) at 1-year	−27.4 (19.2)	33.0 (25.3)	18.5 (13.4)	−0.26 (0.16)	54.2 (47.6)
Effect size	0.88	0.88	0.80	0.88	0.72

Results are displayed as median (and interquartile range)

* *p* < 0.05; ** *p* < 0.01; *** *p* < 0.001; ^ns^ difference not significant – Wilcoxon signed-rank test (all tests compared to baseline)

The number of post-insertion visits for maintenance ranged from 0 to 3 visits per participant, and the overall mean was 1.36 (SD = 1.2). The incidence of prosthodontic events is detailed in Table 5.

**Table 5:** Incidence of prosthodontic events in the 1-year post-treatment period, and the overall prosthodontic outcome (*n* = 15)

	Switzerland (*n* = 6)		Brazil (*n* = 9)		Total (*n* = 15)
	# events	# patients	# events	# patients	# events
Unscheduled visits	3	2	5	3	8
Mandibular implant overdenture					
Matrix activation	5	5	4	3	9
Matrix replacement	1	1	1	1	2
Matrix fixation	0	0	0	0	0
Teeth fracture	0	0	0	0	0
Denture fracture	1	1	0	0	1
Reline	0	0	4	4	4
Denture base adjustment	7	3	10	6	17
Maxillary complete denture					
Teeth fracture	2	2	0	0	2
Denture fracture	0	0	0	0	0
Denture reline	0	0	1	1	1

## Discussion

The novel treatment concept of stabilizing a mandibular complete denture with a single implant in the canine region of the preferred chewing side (c-SIMO) aims to enhance the performance of SIMOs, where, so far, the implant had been exclusively placed in the mandibular midline position. A controlled clinical setting was therefore necessary to determine the viability and feasibility of this treatment option. The findings of this pilot study, which can be considered as preliminary results, indicate that c-SIMOs provide a significant improvement of patient-related outcome measures, and have high implant survival and success rates with minor prosthodontic maintenance and complications, and may therefore be a viable treatment option.

Nevertheless, this study presents with limitations which have to be considered when interpreting the results. The most important shortcoming of this study is the short follow-up time of one year, which is inherent to a pilot study. However, the total absence of peri-implant bone loss and of any implant-related complications during the first year after implant loading is a reassuring result. Various clinical trials on SIMOs have demonstrated very high 5-year implant survival rates in situations where (micro- and moderately) rough implant surfaces were used and a conventional loading protocol was followed [16, 19, 42, 55, 58, 59]. Evidently, no direct comparison to other studies can be made due to the difference in implant positioning, however, the protocols used in this study are in accordance with the parameters that seem to be associated with good long-term survival rates for SIMOs [58]. Therefore, even if a high implant survival is expected for c-SIMOs, the medium- and long-term survival rates are necessary to confirm the viability and success of this treatment modality.

The short observation period in this study may also influence the incidence and type of prosthodontic events reported with this novel treatment option, as it may be a time frame too short to detect complications that could arise during the long-term use of an IOD, such as material fatigue or wear [9]. Multiple studies have demonstrated that the SIMO concept was associated with high rates of denture fracture, as well as a frequent need for matrix replacement/reactivation, possibly due to an overload of the retentive system [16, 20, 22, 23, 36, 41, 59]. Four overdentures required an indirect reline, these were mainly in cases where a surgical osteotomy had been performed for implant placement. The most frequent maintenance events in this study were the adjustment of the denture base and the activation of the matrix. These events were considered normal maintenance events in the short-term follow-up after overdenture treatment. This is in accordance with a previous RCT comparing the prosthodontic maintenance and complications of 1- and 2-IODs over 4 years [24]. It was demonstrated that the majority of the aforementioned maintenance events occurred in the first year, irrespective of the treatment group, and that a longer follow-up did not increase the incidence of the events. Indeed, almost 80% of all denture base adjustments and approximately 50% of matrix activations occurred during the first year [24]. The expected additional maintenance during a longer follow-up may therefore be minimal for c-SIMOs as well.

As mentioned previously, denture fracture has also been reported as a frequent complication associated with SIMOs [16, 23, 36, 59]. Similar rates of this complication can be seen throughout various SIMO studies with no cast framework, with fractures occurring in 20 to over 30% of cases, and even increasing up to 55% in a long-term study [23, 24, 36, 59]. Different distribution patterns of the incidence of denture fractures can be seen, some studies having an increase in the yearly incidence during follow-up, while in another, almost half of the denture fractures occurred during the first year [16, 36]. The preliminary data from the present study shows a low denture fracture rate of less than 7% in one year. Although a very positive finding, it has to be considered that an increase in the incidence of fractures could be seen during a longer follow-up. Nevertheless, these results are in line with the hypothesis that the position of the implant and therefore the retentive element and corresponding housing in the canine region could reduce the risk of denture fracture due to the increased available prosthetic volume in the area compared to the midline.

The significant increase in chewing efficiency and maximum bite force detected in this study demonstrates the functional efficiency of c-SIMOs, despite the short follow-up. The almost two-fold increase in bite force already seen at one year can be attributed to an improved overall function with the c-SIMO, and agrees with previous studies reporting on bite force in patients treated with 2-IODs [12, 21, 50, 69]. The bite force measured in the present study with c-SIMOs seems to be similar to what is reported with midline SIMOs, although a direct comparison is difficult, as there are very limited studies available and evaluation methods differ [7]. The significantly higher bite force on the preferred chewing side (implant side) compared to the contra-lateral side supports the argument that placing the implant close the patient’s chewing center allows for increased bite forces. Nevertheless, after one year of observation, the bite force was similar for both chewing sides. An evaluation after a longer observation period and in a larger cohort is therefore necessary to better understand the influence of the implant position on bite force and overall chewing. This includes the evaluation of the evolution of the preferred chewing side and its potential effect on denture wear and occlusal stability. The significant increase in chewing efficiency detected in the present study in a short observation period is also a very promising finding, as it is not always detected even in studies on overdentures on multiple implants [29, 50]. A direct comparison with other SIMO studies also showing an increase in chewing efficiency cannot be made due to variations in methodology [18, 37, 56].

Last but not least, the patient-reported outcome measures analyzed in the present study also show a significant positive impact of c-SIMOs, with improvements in all tested domains, already present from the first post-insertion visit and persisting throughout the entire follow-up, with almost-perfect scores in all tests at 1 year. The majority of studies on midline SIMOs have also shown marked improvements both in OHRQoL and patient satisfaction [7, 8, 17, 18, 20, 52, 53]. However, there is limited information on PROMs with SIMOs even at medium-term follow-up [16, 20]. More medium- and long-term studies are therefore still necessary to confirm the success of SIMOs in achieving and maintaining high PROMs, and this is evidently the case for c-SIMOs as well.

An additional feature specific to the placement of the implant in the canine area instead of the midline region is the possibility to modify the treatment modality in the future if needed, by placing an additional implant in the contra-lateral canine area. The possibility of placing an additional implant could present a significant further medium- and long-term advantage compared to the midline position, as it maintains more treatment options open for patients according to their specific needs. In this study, none of the patients had requested an additional implant at the end of the observation period, which confirms c-SIMO as a viable minimal-invasive and low-cost treatment modality.

## Conclusions

This pilot study concludes that the single canine-positioned implant for mandibular overdentures (c-SIMO) is a promising treatment option, and may be a viable alternative to the single midline implant overdenture. However, this clinical recommendation must be confirmed in clinical studies designed to evaluate both interventions within the same trial.

## Data Availability

No datasets were generated or analysed during the current study.
